# Extended Exposure to Stiff Microenvironments Leads to Persistent Chromatin Remodeling in Human Mesenchymal Stem Cells

**DOI:** 10.1002/advs.201801483

**Published:** 2018-12-10

**Authors:** Anouk R. Killaars, Joseph C. Grim, Cierra J. Walker, Ella A. Hushka, Tobin E. Brown, Kristi S. Anseth

**Affiliations:** ^1^ Program of Materials Science and Engineering and BioFrontiers Institute University of Colorado Boulder Jennie Smoly Caruthers Biotechnology Building, 3415 Colorado Ave Boulder CO 80303 USA; ^2^ Department of Chemical and Biological Engineering and BioFrontiers Institute University of Colorado Boulder Jennie Smoly Caruthers Biotechnology Building, 3415 Colorado Ave Boulder CO 80303 USA

**Keywords:** allyl sulfide hydrogels, chromatin remodeling, mechanical memory, mesenchymal stem cells, photosoftening

## Abstract

Bone marrow derived human mesenchymal stem cells (hMSCs) are a promising cell source for regenerative therapies; however, ex vivo expansion is often required to achieve clinically useful cells numbers. Recent results reveal that when MSCs are cultured in stiff microenvironments, their regenerative capacity can be altered in a manner that is dependent on time (e.g., a mechanical dosing analogous to a chemical one). It is hypothesized that epigenomic modifications are involved in storing these mechanical cues, regulating gene expression, and ultimately leading to a mechanical memory. Using hydrogels containing an allyl sulfide cross‐linker and a radical‐mediated addition‐fragmentation chain transfer process, in situ softened hMSC‐laden hydrogels at different time points are achieved and the effects of short‐term and long‐term mechanical dosing on epigenetic modifications in hMSCs are quantified. Results show that histone acetylation and chromatin organization adapt rapidly after softening and can be reversible or irreversible depending on time of exposure to stiff microenvironments. Furthermore, epigenetic modulators are differentially expressed depending on the culture history. Collectively, these experiments suggest that epigenetic remodeling can be persistent and might be a memory keeper.

## 1 Introduction

Bone marrow derived human mesenchymal stem cells (hMSCs) are capable of differentiating into multiple lineages, including osteoblasts, adipocytes, and chondrocytes, providing a promising cell source for regenerative therapies;^1^, ^2^ however, in vitro expansion of hMSCs is often necessary to obtain therapeutically relevant cell numbers. During expansion, hMSCs are typically cultured on glassy stiff materials with moduli greater than 1 GPa (e.g., tissue culture polystyrene (TCPS) or glass‐based microbeads) with ill‐defined surface properties. These types of materials can bias cells toward osteogenic differentiation or alter their secretome, thereby decreasing the regenerative properties of transplanted hMSCs.^3^, ^4^, ^5^, ^6^ Recent work has focused more specifically on the effects of substrate stiffness and the role of mechanotransduction on hMSCs, and particularly, how hMSCs sense and integrate mechanical cues from their stiff surroundings that ultimately determines their cell fate.^7^, ^8^, ^9^ For example, hMSCs can remember past mechanical environments, which can influence long‐term cell fate decisions or influence regenerative capacity when transplanted.^10^, ^11^ This mechanical memory has been characterized by irreversible nuclear localization of cotranscription factor Yes‐associated protein (YAP) and depends on the time that hMSCs are cultured on stiff substrates, also called mechanical dosing. A short culture time on stiff substrates (short mechanical dose) results in reversible mechanical memory, in which YAP can translocate from the nucleus to the cytoplasm with nominal changes in gene expression. In contrast, extending the mechanical dose results in irreversible mechanical memory in which YAP persists in the nucleus, causing significant changes in gene expression.^8^, ^10^, ^11^, ^12^, ^13^ It is well‐established that YAP and other transcription factors can influence gene expression; however, it is known that epigenomics can regulate gene expression as well, suggesting its role in mechanical memory.^14^, ^15^, ^16^, ^17^, ^18^, ^19^

Chromatin remodeling primarily regulates gene expression through epigenetic modifications such as acetylation, methylation, and phosphorylation at the amino‐terminal tails of nucleosomal histones.^20^, ^21^, ^22^, ^23^ The acetylation landscape is highly dynamic and governed by two classes of enzymes—histone acetyltransferases (HATs) and histone deacetylases (HDACs). Acetylation of histones by HATs leads to chromatin expansion enabling gene expression.^20^, ^21^, ^22^, ^23^ Alternatively, deacetylation of histones by HDACs results in condensation of chromatin and repression of gene expression.

Previous studies have shown that mechanical cues can influence the epigenomics of hMSCs.^24^, ^25^, ^26^, ^27^, ^28^, ^29^ For example, Heo and co‐workers found that when MSC‐seeded scaffolds were subject to 10% tensile stretch, chromatin condensation increased by ≈80% compared to unloaded controls.^25^ Furthermore, when the scaffolds underwent multiple loading protocols, results revealed higher and sustained levels of condensation, suggesting that previous loading events were imprinted on the nucleus.^24^ Likewise, Downing and co‐workers showed that topology influences epigenomics, as MSCs cultured on 10 µm grooved polydimethylsiloxane (PDMS) surfaces had higher levels of histone 3 acetylation and decreased nuclear HDAC activity compared to smooth surfaces.^30^ Finally, Dahl and co‐workers showed that expanding MSCs on TCPS not only decreases secretory properties and multipotency, but also alters localized genetic and epigenetic modifications, such as increased DNA methylation over time.^31^

Collectively, these data suggest that chromatin remodeling and the epigenetic landscape are likely playing a key role in how hMSCs integrate physical cues from their environment over time. Still, it remains unclear whether these changes in chromatin are reversible, and what role integrated exposure to stiff microenvironments over time (i.e., mechanical dosing) might play in governing these changes. We hypothesized that epigenomic and chromatin remodeling that occurs during expansion of hMSCs will be dependent on the culture history, and that cumulative changes in the chromatin structure over time lead to a cell's mechanical memory.

To isolate the effects of substrate modulus and dynamic changes in the modulus with time, so called mechanical dosing, on hMSCs and chromatin remodeling, we exploited a hydrogel chemistry that allows precise and on‐demand control over the modulus of the material during the culture of hMSCs. Specifically, hydrogels were designed with allyl sulfide crosslinks that undergo an addition‐fragmentation reaction, and thus soften, in response to light via addition of a glutathione thiyl radical.^32^, ^33^ The cytocompatible photochemical reaction is highly efficient, enabling rapid changes in the hydrogel crosslinking density of hydrogels with very low doses of light, allowing us to in situ soften the materials and probe how mechanical dosing affects epigenomics of hMSCs. Using this material, we demonstrate that substrate stiffness can induce changes in chromatin organization by following histone acetylation and condensation and that adaption of these markers occur rapidly after photosoftening. Further, we observed that these changes can be reversible or irreversible depending on the time of exposure to the stiff hydrogel conditions (1 or 10 d, respectively). We identify specific epigenetic modulators that are differentially expressed in these conditions, HAT1, HDAC1, 2, and 3, suggesting a possible mechanism by which chromatin modifications occur. Cumulatively, these experiments suggest that epigenetic modifications and chromatin remodeling contribute to storing mechanical cues in hMSCs, and extended exposure to stiff microenvironments can lead to irreversible changes in the hMSC phenotype and fate determination.

## 2 Results

### 2.1 In Situ Photosoftening Hydrogels Based on Allyl Sulfide Cross‐Linkers

**Figure 1:**
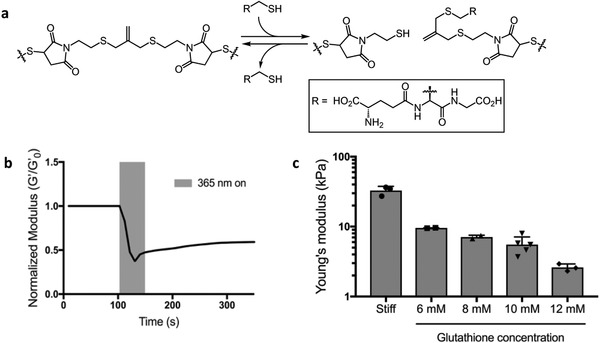
Photo‐mediated rearrangement of allyl sulfide gels and subsequent changes in mechanical properties. a) Mechanism of cross‐linker fragmentation induced via radical‐mediated addition of glutathione to the allyl sulfide cross‐linker. b) Hydrogel was swollen with 12 × 10^−3^ m glutathione and 1.7 × 10^−3^ m LAP and softened by exposing the gel to 365 nm light at 10 mW cm^−2^ for 60 s. The shear storage modulus was tracked and normalized to the initial shear storage modulus (*G*′/*G*′_0_). c) Controlling glutathione concentrations allows for control over degradation. Glutathione was swollen into the network at 0 m (stiff condition), 6, 8, 10, and 12 × 10^−3^ m in the presence of 1.7 × 10^−3^ m LAP and exposed to 10 mW cm^−2^ of 365 nm light for 2 min. This resulted in hydrogels with a Young's modulus of 9.5, 7.1, 5.5, and 2.5 kPa, respectively. The data represent the mean value ± s.d.

To study the influence of mechanical microenvironment and its dynamic changes on epigenetic modifications and chromatin remodeling in hMSCs, we used a hydrogel material chemistry that allows rapid in situ changes in the crosslinking density while maintaining cell–material interactions. Specifically, we synthesized a cross‐linker containing an allyl sulfide functional group, which enables an addition‐fragmentation chain transfer resulting in reversible addition and removal of thiol‐containing molecules. To induce fragmentation, and thus softening, of the hydrogel network, gels were swollen in a buffer solution containing the photoinitiator lithium phenyl‐2,4,6‐trimethylbenzoylphosphinate (LAP) and glutathione. Irradiation with 365 nm light induces photolysis of LAP and generation of glutathione thiyl radicals, which can attack the double bond on the allyl sulfide cross‐linker. The intermediate is unstable and undergoes a β‐scission, resulting in addition of the attacking species, regeneration of the double bond, and conversion of the allyl sulfide crosslinks into a pendant functionality. Subsequent cycles of thiol–ene addition followed by chain transfer from a liberated network thiyl to a free glutathione replace crosslinking allyl sulfides with their non‐crosslinked counterparts, thus softening the network (**Figure** **1**a). Thiyl–thiol chain‐transfer renders this process highly efficient and leads to fast degradation rates due to one initiation event leading to multiple cross‐linking cleavage events.^32^

hMSCs are often used as a model to study mechanosensing. In stiff microenvironments (*E* > 10 kPa), hMSCs have been shown to become biased toward osteogenic differentiation, often accompanied by YAP localization in the nucleus.^5^ In contrast, culturing hMSCs on soft hydrogels (*E* < 10 kPa) maintains multipotency, and YAP remains largely distributed in the cytoplasm. Therefore, the stiff, osteogenic promoting condition was chosen to be above *E* = 10 kPa and soft, deactivating condition was chosen to be below *E* = 10 kPa.

Gels were formed by copolymerizing eight‐arm 20 kDa PEG thiol (40 × 10^−3^ m thiol) with the allyl sulfide dimaleimide cross‐linker (44 × 10^−3^ m maleimide) through an anionic step growth reaction initiated by triethanolamine (TEOA). The gel point was reached within 30 s and a final shear storage modulus (*G*′) of 12 kPa or Young's modulus (*E*) of 36 kPa was achieved within 10 min (Figure S1, Supporting Information). The viscous modulus (*G*″) was consistently several orders of magnitude lower than the storage modulus confirming the gel‐like state (Figure S1, Supporting Information). The magnitude of change in the gel modulus can be finely tuned by controlling the glutathione concentration introduced into the network.^32^ Here, we varied the concentration from 6 to 12 × 10^−3^ m glutathione while keeping the LAP concentration (1.7 × 10^−3^ m) constant and irradiated with 365 nm light (*I*_0_ = 10 mW cm^−2^) for 2 min to generate soft conditions with *E* < 10 kPa.^10^ The network rearrangement of a hydrogel swollen with 12 × 10^−3^ m glutathione and 1.7 × 10^−3^ m LAP was tracked during light exposure by monitoring the shear storage modulus over time (Figure 1b). The final equilibrium swollen Young's modulus can be seen in Figure 1c compared to the stiff condition (*E* = 32.7). Introduction of 6, 8, 10, and 12 × 10^−3^ m glutathione resulted in hydrogels with a Young's modulus of 9.5, 7.1, 5.5, and 2.5 kPa, respectively.

### 2.2 Chromatin Remodeling in hMSCs Depends on Microenvironmental Stiffness

We first investigated if the mechanical microenvironment could influence chromatin remodeling. hMSCs were cultured on allyl sulfide functionalized PEG hydrogels with varying moduli (*E* = 32.7, 9.5, 7.1, 5.5, and 2.5 kPa) for 3 d, and the YAP nuclear‐to‐cytoplasm (Nuc/Cyt) ratio was analyzed on these substrates. All results were compared to hMSC cultured on stiff, osteogenic promoting condition (*E* = 32.7 kPa) to identify the softened gels for dynamic experiments (Figure S2, Supporting Information). Based on YAP Nuc/Cyt ratio, cell and nucleus morphology the 5.5 kPa hydrogel was chosen as soft, deactivating condition for subsequent experiments, as it is not significantly different from the 2.5 kPa condition and supports better hMSC attachment.

**Figure 2:**
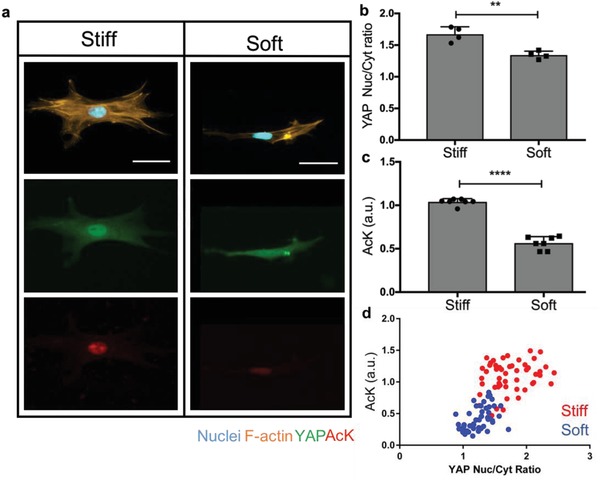
Influence of substrate modulus on YAP nuclear localization and AcK modification. a) Immunostaining of hMSCs on soft (*E* = 5.5 kPa) and stiff (*E* = 32.7 kPa) hydrogel surfaces after a 3 d culture. hMSCs cultured on the stiff substrate show YAP nuclear localization and high levels of histone acetylation (AcK) staining. hMSCs cultured on the soft substrate show cytoplasmic YAP localization and low levels of AcK staining. Nucleus (blue), F‐actin (orange), YAP (green), and AcK (red). Scale bars = 50 µm. b) YAP nuclear to cytoplasm (Nuc/Cyt) ratio was quantified based on immunostaining. hMSCs on stiff substrates showed a significant increase in YAP Nuc/Cyt ratio. **: *p* < 0.01, based on *t*‐test. *n* = 4 with more than 100 hMSCs analyzed per sample. The data represent the mean value ± s.d. c) Relative AcK intensity was quantified based on immunostaining. hMSCs on stiff substrates showed a significant increase in AcK intensity. ****: *p* < 0.0001, based on *t*‐test. *n* = 7 with more than 100 hMSCs analyzed per sample. The data represent the mean values ± s.d. d) YAP nuclear‐to‐cytoplasm ratio is significantly positively correlated to histone acetylation intensity in hMSCs cultured on stiff and soft substrates. Pearson correlation coefficient = 0.7, *p* < 0.0001. 50 cells per condition plotted.

Next, hMSCs were cultured on stiff (*E* = 32.7 kPa) and soft (*E* = 5.5 kPa) hydrogels for 3 d and histone acetylation levels were quantified by immunostaining. hMSCs on the stiff substrate where YAP nuclear localization is high, also show high levels of histone acetylation (AcK) staining. In contrast, hMSCs cultured on the soft substrate where YAP is primarily cytoplasmic show low levels of AcK staining (**Figure** **2**a). Quantification of the immunostaining demonstrates a significant increase in YAP Nuc/Cyt ratio in hMSCs in the stiff condition compared to the soft condition, consistent with prior results^10^ (Figure 2b). Interestingly, histone acetylation intensity was also significantly increased in hMSCs on stiff hydrogels compared to soft substrates, indicating that histone acetylation can be influenced by substrate moduli (Figure 2c). Histone acetylation immunostaining was verified by Western blot (Figure S3, Supporting Information). Finally, we examined the relationship between the YAP Nuc/Cyt ratio and histone acetylation intensity (Figure 2d) for the hMSCs culture conditions, and two distinct clusters were observed, corresponding to the stiff or soft substrates. A significant positive correlation with Pearson correlation coefficient of 0.7 was measured, meaning that when the YAP Nuc/Cyt ratio increases, histone acetylation in the same cell increases as well.

**Figure 3:**
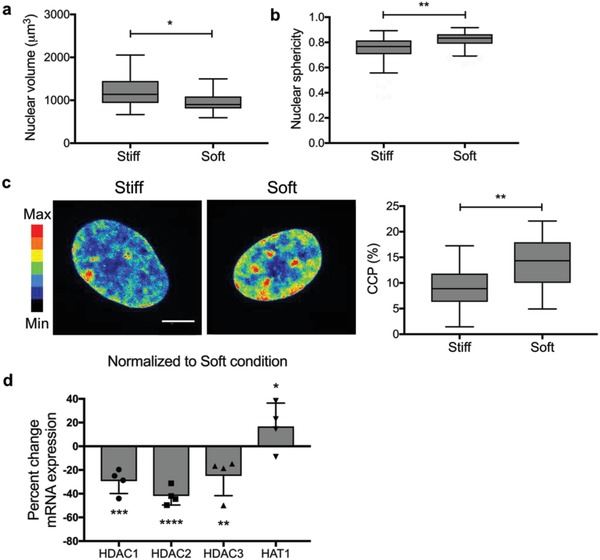
Influence of substrate modulus on nuclear morphology, chromatin condensation, and mRNA expression of epigenetic modulators. a) Nuclear volume was quantified based on nuclear immunostaining with DAPI after a 3 d culture. Nuclear volume was significantly larger for hMSCs cultured on stiff substrates compared to soft substrates. *: *p* < 0.05 based on *t*‐test. *n* = 3 with more than 20 hMSCs analyzed per sample. Values are shown as median ± 1.5 IQR. b) Nuclear sphericity was quantified based on nuclear immunostaining with DAPI after a 3 d culture. Nuclear sphericity was significantly larger for hMSCs cultured on soft substrates. **: *p* < 0.01 based on *t*‐test. *n* = 3 with more than 20 hMSCs analyzed per sample. Values are shown as median ± 1.5 IQR. c) Chromatin condensation was visualized by creating a heatmap of the DAPI intensity and calculating the chromatin condensation parameter (CCP) using a MATLAB script. Nuclei on soft substrates showed overall higher DAPI intensity and higher intensity clusters within the nucleus compared to the nuclei on stiff substrates. CCP values for nuclei on soft substrates were significantly larger, indicating more chromatin condensation in nuclei on soft substrates compared to nuclei cultured on stiff substrates. **: *p* < 0.01 based on *t*‐test. *n* = 3 with more than 20 hMSCs analyzed per sample. Values are shown as median ± 1.5 IQR. Scale bar = 10 µm. d) RT‐qPCR was performed and mRNA expression on stiff substrates was normalized to mRNA expression on soft substrates. Expression of HDAC1, HDAC2, and HDAC3 is significantly decreased in hMSCs on stiff substrates compared to soft substrates after a 3 d culture, whereas HAT1 expression was increased. *: *p* < 0.05, **: *p* < 0.01, ***: *p* < 0.001, ****: *p* < 0.0001 based on one‐way ANOVA followed by Tukey's post hoc test. *n* = 4 with triplicates. The data represent the mean value ± s.d.

Based on the observation that increased histone acetylation occurs on stiff hydrogels compared to soft hydrogels, we sought to investigate how nuclear shape and volume, potential markers of chromatin remodeling, are altered by substrate stiffness. The extracellular matrix (ECM) is directly connected to the nucleus via attachment of the cytoskeleton to LINC complexes on the nuclear membrane,^34^, ^35^ and hMSCs interactions on stiff substrates can lead to cytoskeletal tension that exerts external forces on the nucleus, causing its deformation. Indeed, nuclear deformation has been shown to induce changes in chromatin organization that results in changes in gene expression.^34^, ^36^ Hence, we characterized the morphology of hMSC nuclei when cultured on stiff and soft substrates. After 3D reconstruction of confocal images, the nuclear volume and sphericity were calculated (**Figure** **3**a,b), nuclear volume was significantly larger on stiff substrates compared to soft substrates. Moreover, nuclear sphericity was significantly lower on stiff substrates compared to soft substrates.

We hypothesized that the observed increase in nuclear volume and decrease in sphericity in hMSCs cultured on stiff substrates, could be due to a decrease in chromatin condensation. Additionally, this would correlate to the increase in histone acetylation levels observed in hMSCs during exposure to the stiff conditions. Therefore, further studies were completed to ascertain levels of chromatin condensation by analyzing DAPI staining intensity and calculating a chromatin condensation parameter (CCP) using a MATLAB script.^37^ From representative heatmaps of DAPI intensity of nuclei on stiff and soft substrates, we observe that nuclei on soft substrates showed overall higher DAPI intensity and more clusters within the nucleus compared to the nuclei on stiff substrates (Figure 3c). The calculated CCP values for both conditions show striking differences, where nuclei on soft substrates have significantly larger CCP values. This result supports the conclusion that chromatin is more condensed in nuclei on soft substrates compared to nuclei on stiff substrates.

### 2.3 HDACs and HAT1 Play a Role in Stiffness Induced Chromatin Remodeling

Chromatin condensation and histone acetylation are regulated by epigenetic modulators, including HATs and HDACs. To investigate if the increase in histone acetylation and decrease in chromatin condensation seen in hMSCs cultured on stiff substrates is caused by differential expression of these enzymes, we performed quantitative real‐time polymerase chain reaction (qRT‐PCR) to measure mRNA expression of HDAC1, HDAC2, HDAC3, and HAT1 on stiff and soft substrates. We observed that expression of HDAC1, HDAC2, and HDAC3 is significantly decreased in hMSCs on stiff substrates compared to soft substrates after 3 d of culture (Figure 3d). In addition, immunostaining revealed that global and nuclear levels of HDAC3 were significantly higher in hMSCs cultured on soft substrates (Figure S4, Supporting Information). HAT1 expression was increased at this time point (Figure 3d). These data correlate to the increased histone acetylation levels observed in hMSCs on stiff substrates and to the lower CCP values (Figures 2c and 3c). Overall, substrate stiffness affects the chromatin architecture—stiff substrates induce increased histone acetylation through decreased expression of HDACs and higher expression of HATs—ultimately leading to a more open chromatin structure, but we were interested in how these chromatin modifications would change over time.

### 2.4 Chromatin Remodeling after Substrate Softening Is Rapid

**Figure 4:**
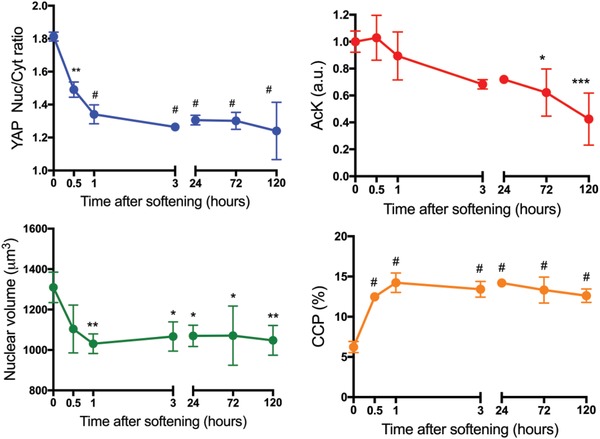
Dynamics of YAP, histone acetylation, nuclear volume, and chromatin condensation after softening. hMSCs were cultured on stiff substrates (*E* = 32.7 kPa) for 1 d and subsequently in situ softened by irradiation with 10 mW cm^−2^ 365 nm light for 2 min (*E* = 5.5 kPa). YAP Nuc/Cyt ratio, AcK levels, nuclear volume, and CCP were quantified based on immunostaining prior to softening and at multiple time points after softening. *: *p* < 0.05, **: *p* < 0.01, ***: *p* < 0.001, #: *p* < 0.0001, compared to *t* = 0 condition, based on one‐way ANOVA followed by Tukey's post hoc test. For YAP and AcK, *n* = 3 with more than 100 hMSCs analyzed per sample. For nuclear volume and CCP, *n* = 3 with more than 20 hMSCs analyzed. The data represent the mean value ± s.d.

The previous findings indicate that chromatin remodeling and epigenetic modifications are influenced by the magnitude of the substrate modulus (*E* = 32.7 kPa, stiff, and *E* = 5.5 kPa, soft). Next, we sought to probe the dynamics of the adaption process of histone acetylation, nuclear volume, and chromatin condensation after the substrate modulus was softened. We hypothesized that chromatin remodeling would adapt in a rapid fashion to soft basal levels, as soon as the cytoskeletal tension was disintegrated. Therefore, hMSCs were cultured on stiff substrates (*E* = 32.7 kPa) for 1 d and subsequently in situ softened with light to a final *E* = 5.5 kPa. Control experiments confirmed that in situ softening with 10 × 10^−3^ m glutathione and 1.7 × 10^−3^ m LAP did not have any influence on YAP localization, histone acetylation levels, and CCP (Figure S5, Supporting Information) and that the Young's modulus of the substrate remained 5.5 kPa after light‐induced softening for the time course of the following experiments (Figure S6, Supporting Information). Chromatin remodeling was assessed by quantification of YAP Nuc/Cyt ratio, histone acetylation levels, nuclear volume, and CCP prior to softening and at multiple time points post substrate softening (**Figure** **4**). As expected, YAP moves from the nucleus to the cytoplasm rapidly, and a significant difference can be observed as early as 0.5 h after softening. Nuclear volume and CCP also change rapidly; chromatin condensation is significantly increased as early as 0.5 h after softening and nuclear volume is significantly decreased 1 h after softening. While YAP localization and chromatin condensation change in the first few hours and then stabilize, histone acetylation levels decrease slower and over a longer time period. Significantly lower histone acetylation levels are observed after 72 h post softening, which may indicate that not only cytoskeletal tension influences histone acetylation, but also upregulation of HDACs to remove histone acetylation may be a slower step. This hypothesis was further supported by disruption of the cytoskeletal tension with blebbistatin (Figure S7, Supporting Information). The next question that we asked was if the chromatin changes remain reversible if the time on stiff substrates was extended.

### 2.5 Chromatin Remodeling in hMSCs Depends on Mechanical Dosing

**Figure 5:**
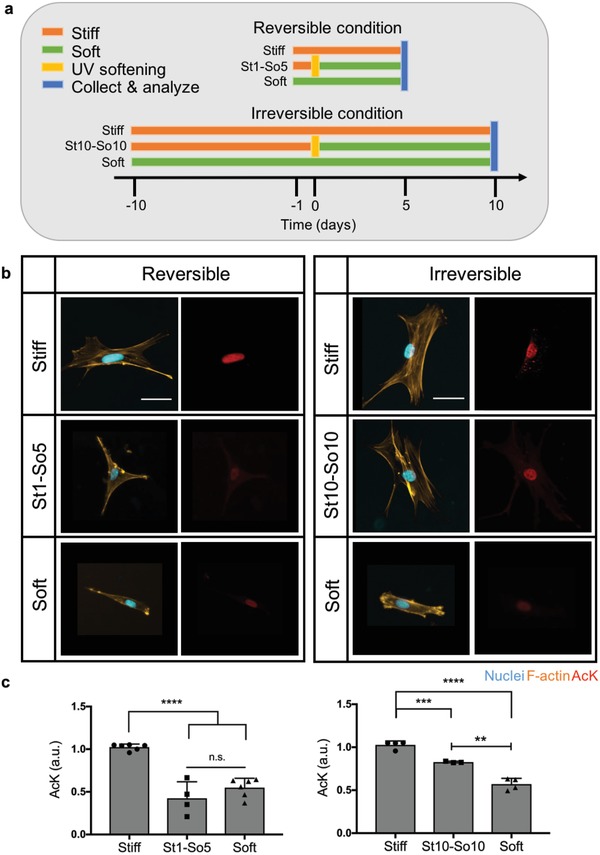
Influence of reversible and irreversible mechanical dosing on AcK modification. a) Experimental outline of reversible and irreversible condition based on YAP staining. *E* = 32.7 kPa for stiff and *E* = 5.5 kPa for soft hydrogel conditions. For the reversible condition, hMSCs were cultured on stiff hydrogel substrates (orange) for 1 d. Subsequently, hydrogels were in situ softened by irradiation with 10 mW cm^−2^ 365 nm light for 2 min (yellow) and hMSCs were cultured for an additional 5 d on soft substrates (green) before collection and analysis (blue) (St1‐So5). For the irreversible condition, hMSCs were cultured on stiff hydrogel substrates for 10 d. Subsequently, hydrogels were in situ softened by irradiation with 10 mW cm^−2^ 365 nm light for 2 min and hMSCs were cultured for an additional 10 d on soft substrates before collection and analysis (St10‐So10). Both the reversible and irreversible conditions have controls of hMSCs cultured on hydrogel substrates with constant stiff (*E* = 32.7 kPa) or soft (*E* = 5.5 kPa) moduli. b) Immunostaining of hMSCs cultured in a reversible or irreversible condition compared to their stiff and soft control conditions. hMSCs cultured in the condition that leads to reversible YAP signaling (St1‐So5) showed less AcK staining compared to the stiff control condition, but similar AcK staining as the soft control condition. hMSCs cultured in the condition that leads to irreversible YAP nuclear localization (St10‐So10) showed brighter AcK staining compared to the soft control condition, but less AcK staining compared to the stiff control condition. Nucleus (blue), F‐actin (orange), and AcK (red). Scale bars = 50 µm. c) Relative AcK intensity was quantified based on immunostaining for both reversible and irreversible condition. Softening after 1 d (St1‐So5 condition) allowed AcK levels to revert to basal levels for the soft condition. Softening after 10 d (St10‐So10 condition) resulted in AcK levels significantly above basal levels for the soft condition. n.s.: *p* > 0.05, **: *p* < 0.01, ***: *p* < 0.001, ****: *p* < 0.0001 based on a one‐way ANOVA followed by Tukey's post hoc test. *n* ≥ 3 with more than 100 hMSCs analyzed per sample. The data represent the mean value ± s.d.

We were curious whether histone modifications, such as acetylation and chromatin condensation, are integrated over time and might be implicated in the development of a mechanical memory in hMSCs. To test this notion, we cultured hMSCs on stiff hydrogels (*E* = 32.7 kPa) and at prescribed time points, in situ softened the substrates with light to a final *E* = 5.5 kPa. Previous studies from our lab have identified times of exposure of hMSCs to stiff microenvironments (1–10 d) that can lead to reversible and irreversible YAP and cbfa‐1 localization to the nucleus, which ultimately influenced MSC fate.^10^ We confirmed that similar time lines yielded reversible or irreversible nuclear YAP in hMSC with the allyl sulfide PEG hydrogel chemistry and softening conditions (Figure S8, Supporting Information). Specifically, a mechanical dose of 1 d on stiff hydrogels (St1) followed by 5 d on soft hydrogels (So5) after in situ softening (St1‐So5) afforded reversible YAP nuclear localization, while a mechanical dose of 10 d on stiff hydrogels (St10) followed by 10 d on soft hydrogels (So10) after in situ softening (St10‐So10) afforded irreversible YAP nuclear localization. hMSCs were also cultured exclusively on stiff or soft hydrogel substrates as control experiments (**Figure** **5**a).

These time lines (Figure 5a) were subsequently used to investigate chromatin remodeling. As shown by representative images of histone acetylation staining for the reversible mechanical dosing condition (Figure 5b), histone acetylation levels in the St1‐So5 condition resemble the soft control hMSCs. In contrast, for the irreversible mechanical dose, histone acetylation staining of hMSCs in the St10‐So10 condition resembles the stiff control hMSCs (Figure 5b). Quantitative analysis reveals that histone acetylation levels in the reversible condition decreased after 5 d on softened hydrogels back to the baseline acetylation observed on control soft hydrogels (Figure 5c). However, histone acetylation levels in the irreversible condition remained significantly above baseline acetylation observed on soft hydrogels, even 10 d post softening. These data demonstrate that the reversibility of histone acetylation is dependent on the mechanical dose hMSCs receive: histone acetylation seems to be reversible with a St1‐So5 mechanical dose in contrast to a St10‐So10 mechanical dose, where histone acetylation does not revert to soft basal levels. This indicates that mechanical memory might not only be instilled by persistent nuclear YAP localization, but also by persistent histone acetylation.

To verify that there were differences between stiff and soft conditions after a mechanical dose of 1 and 10 d, histone acetylation levels were analyzed after hMSCs were cultured on stiff hydrogels for 1 and 10 d. We observed that histone acetylation levels were significantly increased on stiff substrates as soon as day 1 and that the levels remained significantly increased until day 10 (Figure S9, Supporting Information).

**Figure 6:**
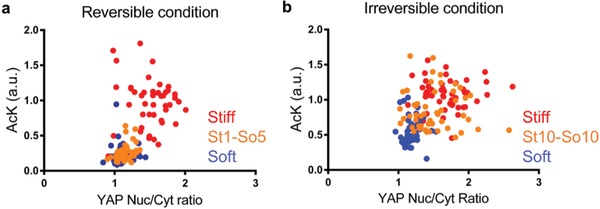
Significant positive correlation between YAP Nuc/Cyt ratio and histone acetylation for hMSCs grown on hydrogels in both reversible and irreversible conditions. a) Single cell scatter plot of histone acetylation intensity as a function of YAP Nuc/Cyt ratio for hMSCs in stiff (red), soft (blue), or St1‐So5 (orange) conditions. The population of hMSCs cultured in the St1‐So5 condition (orange) cluster with the population of hMSCs cultured in the soft control condition (blue). b) Single cell scatter plot of histone acetylation intensity as a function of YAP Nuc/Cyt ratio for hMSCs in stiff (red), soft (blue), or St10‐So10 (orange) conditions. The population of hMSCs cultured in the St10‐So10 condition cluster more with the population of hMSCs cultured in the stiff control condition (red).

Additionally, we investigated the relationship between YAP Nuc/Cyt ratio and histone acetylation intensity for the reversible and irreversible conditions (**Figure** **6**). There was a significant trend correlating greater YAP Nuc/Cyt ratio with higher histone acetylation intensity for hMSCs in both reversible and irreversible conditions with a Pearson correlation coefficient of 0.61 and 0.42, respectively. Distinct populations are evident in both scatter plots: in the reversible condition, the population of hMSCs cultured in the St1‐So5 condition, clustered toward the population of hMSCs cultured in the soft control condition (Figure 6a). Whereas in the irreversible condition, hMSCs cultured in the St10‐So10 condition moved toward the population of hMSCs cultured in the stiff control condition (Figure 6b).

**Figure 7:**
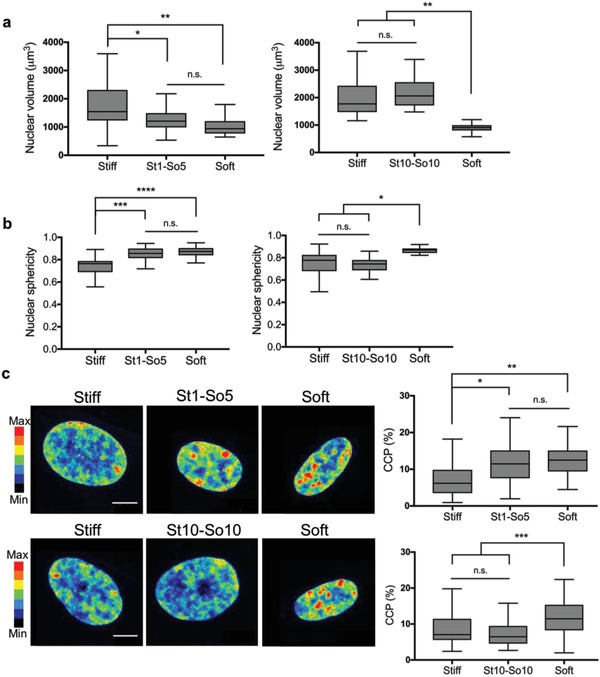
Influence of reversible and irreversible mechanical dosing on nuclear morphology and chromatin condensation. a) Nuclear volume was quantified based on nuclear immunostaining with DAPI for reversible or irreversible condition. Softening after 1 d (St1‐So5 condition) allowed nuclear volume to revert to basal levels for the soft condition. Softening after 10 d (St10‐So10 condition) resulted in nuclear volume significantly above basal levels for the soft condition but comparable to levels of the stiff condition. n.s.: *p* > 0.05, *: *p* < 0.05, **: *p* < 0.01 based on one‐way ANOVA followed by Tukey's post hoc test. *n* = 3 with more than 20 hMSCs analyzed per sample. Values are shown as median ± 1.5 IQR. b) Nuclear sphericity was quantified based on nuclear immunostaining with DAPI for reversible or irreversible condition. Softening after 1 d (St‐So5 condition) allowed nuclear sphericity to revert to basal levels for the soft condition. Softening after 10 d (St10‐So10 condition) resulted in nuclear sphericity comparable to the stiff condition but significantly above basal levels for the soft condition. n.s.: *p* > 0.05, *: *p* < 0.05, ***: *p* < 0.001 based on one‐way ANOVA followed by Tukey's post hoc test. *n* = 3 with more than 20 hMSCs analyzed per sample. Values are shown as median ± 1.5 IQR. c) Chromatin condensation was visualized by creating a heatmap of the DAPI intensity and calculating the chromatin condensation parameter (CCP) using a Matlab script. Nuclei in the St1‐So5 condition showed high intensity clusters within the nucleus comparable to the nuclei on the soft condition. CCP values for this condition reverted to basal levels for the soft condition. Nuclei in the St10‐So10 condition showed less high intensity clusters comparable to nuclei on the stiff condition. CCP values for this condition remained above basal levels for the soft condition but comparable to levels of the stiff condition. n.s.: *p* > 0.05, *: *p* < 0.05, ***: *p* < 0.001 based on one‐way ANOVA followed by Tukey's post hoc test. *n* = 3 with more than 20 hMSCs analyzed per sample. Values are shown as median ± 1.5 IQR. Scale bar = 10 µm.

Observing that histone acetylation can be transient or persistent depending on mechanical dosing, we next examined the nuclear volume and sphericity of hMSCs cultured under these same conditions (**Figure** **7**a,b). Results reveal that the nuclear volume for hMSCs in the St1‐So5 condition was significantly smaller compared to the nuclear volume for hMSCs on the stiff hydrogel control, but similar to the soft hydrogel control (Figure 7a). These data suggest that the nuclei volume in the St1‐So5 condition decreased after in situ softening, reverting to a state similar to cells exposed only to soft microenvironments. Nuclear sphericity followed the same trend, where the nuclei in the St1‐So5 condition increased their sphericity after in situ softening, reversing to levels observed in soft hydrogel controls (Figure 7b). In contrast, nuclear volume and nuclear sphericity for hMSCs in the St10‐So10 condition persisted at stiff control values after in situ softening, even after 10 d on soft microenvironments, demonstrating that the cells did not respond to the decrease in modulus.

In addition to nuclear morphology, chromatin condensation was analyzed by DAPI staining and CCP values were quantified. DAPI intensity is higher in nuclei cultured in the St1‐So5 condition compared to the St10‐So10 condition as shown by representative heatmaps of DAPI intensity (Figure 7c). Furthermore, nuclei in the St1‐So5 condition resemble the soft hydrogel control whereas the nuclei in the St10‐So10 condition resemble the stiff hydrogel control. The CCP values show this same trend, indicating that not only histone acetylation, but also chromatin condensation is reversible in the St1‐So5 condition with a short mechanical dose, but irreversible in the St10‐So10 condition with a long mechanical dose.

### 2.6 HDACs and HAT1 Play a Role in Reversible and Irreversible Chromatin Remodeling

**Figure 8:**
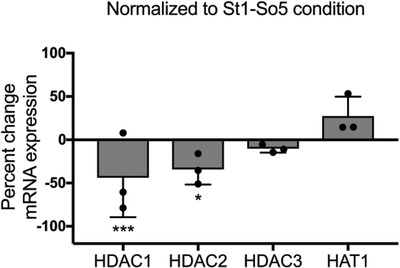
Influence of reversible and irreversible mechanical dosing on mRNA expression of epigenetic modulators. RT‐qPCR was performed and mRNA expression in the St10‐So10 condition was normalized to mRNA expression in the St1‐So5 condition. Expression of HDAC1, HDAC2, and HDAC3 is significantly decreased in hMSCs during irreversible mechanical dosing compared to reversible dosing, whereas HAT1 expression was increased. *: *p* < 0.05, ***: *p* < 0.001 based on one‐way ANOVA followed by Tukey's post hoc test. *n* = 3 with triplicates. The data represent the mean value ± s.d.

To examine if the reversibility and irreversibility of chromatin remodeling is related to differential expression of epigenetic modulators, we performed qRT‐PCR to measure mRNA expression of HDAC1, HDAC2, HDAC3, and HAT1 in hMSCs dosed with a short or long mechanical dose. The percent change in mRNA expression of these modulators in the St10‐So10 condition normalized to the St1‐So5 condition was quantified (**Figure** **8**). We observed that hMSCs in the St10‐So10 condition express significantly lower HDAC1 and HDAC2 compared to hMSCs in the St1‐So5 condition, while HAT1 seems to be more highly expressed in the St10‐So10 condition. Furthermore HDAC1, HDAC2, and HDAC3 remained upregulated in soft control conditions and HAT1 remained upregulated in stiff control conditions (Figure S10, Supporting Information). These results correlate to the reversibility of histone acetylation levels, nuclear morphological changes, and CCP values for hMSCs dosed with a short mechanical dose and to the irreversibility of histone acetylation levels, nuclear morphological changes, and CCP values observed in hMSCs dosed with a long mechanical dose.

## 3 Discussion

Human MSCs hold significant promise for regenerative therapies as indicated by their involvement in an increasing number of clinical trials each year. For most applications, it is essential to expand hMSCs in vitro to achieve a therapeutically relevant cell number prior to transplantation. Results have shown that this expansion process can decrease the regenerative properties of hMSCs,^3^, ^6^, ^38^ but less is known about how the culture history can be influential over time to induce a mechanical memory. In this study, we were particularly interested in the mechanosensitivity of hMSCs, and using a photoadaptable hydrogel system to systematically investigate epigenetic changes that may occur as a result of the modulus of the culture microenvironment. Ultimately, this work aimed to study the mechanism behind mechanical memory in depth, using highly controlled hydrogel materials, to elucidate pathways that could be blocked or alternative substrates that could be used to promote long‐term hMSC expansion without altering their regenerative phenotype.

Herein, we designed a hydrogel culture platform that allows precise and in situ control over the gel modulus. We developed an allyl sulfide cross‐linker that enables rapid hydrogel softening via a radical‐mediated addition‐fragmentation chain‐transfer reaction (Figure 1a). Previously, our lab has relied on hydrogels crosslinked with a nitrobenzyl linker that can undergo photolysis, and thus soften, to explore cellular mechanotransduction.^10^, ^13^, ^39^ A key limitation with nitrobenzyl hydrogels is their slow rate of photodegradation due to each photolysis reaction requiring absorption of a photon. Since the allyl sulfide reaction is radical‐mediated, absorption of a photon by LAP can lead to many subsequent fragmentation reactions, greatly increasing the rate of hydrogel degradation.^32^ Thus, we were able to soften stiff hydrogels (*E* = 32.7–5.5 kPa) with only 2 min of irradiation (Figure 1b,c), which would otherwise not be possible using the nitrobenzyl strategy. Our new approach allowed us to study how hMSCs respond to changes in their local microenvironment, and design experiments to test mechanical dosing effects on the epigenomics of hMSCs. Our results suggest that persistent chromatin remodeling might play an important role in establishing a mechanical memory in hMSCs over time. Initial results show that chromatin remodeling in hMSCs happens consequent to mechanical cues. Histone acetylation levels increase significantly, and the chromatin condensation parameter strikingly decreases in a stiffness‐dependent manner (Figures 2 and 3).

These observations further correlate to the expression of epigenetic modulators, as measured through elevated mRNA expression of HAT1 and a lower mRNA expression of HDAC1, HDAC2, and HDAC3 in hMSCs cultured on stiff hydrogels (Figure 3d). Collectively, these results indicate that chromatin is opened by histone acetylation by HAT1 when hMSCs are cultured on stiff substrates, while chromatin is condensed by histone deacetylation by HDACs when hMSCs are cultured on soft substrates. These results follow trends during osteogenic differentiation of hMSCs where HDAC1 expression decreases and histone acetylation increases at promoter sites of osteogenic genes, such as osteocalcin.^23^, ^40^

In situ softening of the hydrogel modulus enabled us to study the time dynamics of chromatin remodeling. Chromatin remodeling adapted in a rapid fashion to the softened substrate (Figure 4). YAP diffused from the nucleus to the cytoplasm as early as 0.5 h after softening, consistent with other diffusion times published.^41^ Nuclear volume and CCP also adapted rapidly, however interestingly histone acetylation decreased slower and over a longer time period. These data suggest that histone acetylation is not only influenced by cytoskeletal tension but by an interplay of physical cues and protein signaling.

Next, we hypothesized that epigenomic changes may be occurring when hMSCs are expanded or cultured using traditional substrates that are very stiff (e.g., tissue culture plastic, glass beads), and that exposure to these environments can lead to long‐term effects that influence therapeutic outcomes. For brevity, we refer to these integrated effects that relate to the mechanics of the substrates as a mechanical memory. Our results reveal that nuclear morphology, histone acetylation, and chromatin condensation are reversible when the time of exposure to stiff substrates is short (i.e., as low mechanical dose), but the effects can become irreversible with extended exposure and dosing (Figures 5, 6, 7). Nuclear morphology and CCP levels remain at stiff control levels, while histone acetylation decreases to levels in between its stiff and soft control. Although most key parameters remain irreversible, it is possible that histone acetylation levels decrease further when cultured on soft for an extended period. Moreover, HDACs and HAT1 appear to play a role in the (ir)reversibility of chromatin remodeling (Figure 8). Expression of HDAC1 and HDAC2 is significantly downregulated and expression of HAT1 appears to be upregulated when the hMSCs lose their ability to revert to soft basal levels. These expression levels are similar to the stiff basal levels, indicating that expression levels of HAT1 and HDACs can become irreversible. Irreversible changes in the epigenome, such as histone acetylation, can depend on the continued expression of HAT1 and restrained expression of HDAC 1 and 2 after the mechanical dosing had stopped. This notion further suggests that protein signaling is also involved besides physical tension within the cell.

These results give some insight as to how mechanical extracellular cues are directly converted into nuclear biochemical signals over time. Taken together, persistent nuclear YAP localization (and other transcription factors, such as cbfa1), suggest epigenetic changes are occurring in hMSC, such as persistent chromatin remodeling, histone acetylation, and persistent up‐ and downregulation of epigenetic modulators. Due to epigenetic persistence, such as persistent upregulation of HAT1, hMSCs might lose their mechanosensitivity (i.e., reversible response to changes in stiff‐to‐soft microenvironments) This implicates that on an epigenetic level, hMSCs are already committed to a lineage and therefore are not able to respond to mechanical change anymore.

The timing of this persistence process might be variable, as cells likely integrate cues across their culture history or exposure to microenvironmental cues. Here, our conclusions are based on the relation between the St1‐So5 and St10‐So10 conditions and their stiff and soft control groups, a particular time period of MSC culture on highly controlled hydrogel matrices. However, one must note that there was an expansion of the hMSC to P2, specifically 10 d of culture on TCPS, prior to the initiation of these studies. This expansion was included to isolate the cells from the bone marrow aspirate, but could influence the timing of the mechanical memory. Would early and late passage hMSCs respond on the same time scale? Rao et al. investigated the influence of passaging on hMSCs mechanosensitivity and showed that up to P7 YAP shuttled to the cytoplasm when transferred to soft hydrogels.^6^ Others have studied the influence of repetitive switching between different mechanical environments, such as stretching or passaging between glass and a 20 kPa substrate, on chromatin remodeling.^24^, ^42^ Vautier and co‐workers demonstrate that increased passaging between glass and a 20 kPa substrate decondenses the chromatin.^42^ This raises the question as to whether it is the cumulative time on a stiff substrate that induces this epigenetic change or the time span of each repetition, that is, passages. Both serial expansion and static exposure to stiff cues can induce mechanical memory in stem cells, so the relationship between the integral of time on stiff and the corresponding mechanical memory is variable and not well understood. These repetitions could modify the timing of epigenetic changes and mechanical memory as well and are important to consider when designing experiments and interpreting results.

While our work links persistent chromatin remodeling to mechanical memory induced by long‐term culture on stiff hydrogel substrates (mechanical dosing), other papers have associated microRNA levels (especially miR‐21) with mechanical memory in hMSCs.^11^, ^43^ Hinz and co‐workers demonstrated that miR‐21 is a mechanical memory keeper as levels remained high long after stiff priming.^11^ Taking our results together, mechanical cues can induce permanent changes at the protein level, micro RNA level, and epigenome. Thus, hMSCs experience a complex interplay of all these persistent changes at different levels within the cell that establish a mechanical memory and ultimately determines the fate of hMSCs.

Future work will focus on performing more precise measures of how the epigenome of hMSCs changes with a reversible or irreversible mechanical dose. For instance, specific modifications can be analyzed, such as acetylation of H3K9 or tri‐methylation of H3K4, two modifications that are commonly associated with gene activation. Furthermore, changes in repressive markers can be examined, such as tri‐methylation of H3K27, a modification commonly associated with gene repression.^44^ Once specific markers are associated with an irreversible phenotype, genes with these markers can be identified using chromatin immunoprecipitation‐sequencing (ChiP‐Seq), linking mechanically induced changes in epigenetic modifications to fate determination.

Our experiments provided some new insight in the pathways that might contribute to mechanical memory development and maintenance. In the end, more efficient ways to regulate and maintain multipotency during cell expansion in vitro can be established that are highly beneficial for therapeutic applications.

## 4 Conclusion

In summary, we used a phototunable PEG‐based hydrogel system that allowed us to soften the matrix modulus on demand to study the effect of the culturing history on the epigenomics of hMSCs. Culturing hMSCs on stiff and soft hydrogel substrates revealed that chromatin remodeling happens as a consequence to mechanical cues. Histone acetylation was increased, and chromatin condensation was decreased in hMSCs cultured on stiff hydrogels substrates. These epigenetic differences further correlate to the expression of epigenetic modulators, with elevated mRNA expression of HAT1 and a lower mRNA expression of HDAC1, HDAC2, and HDAC3 in hMSCs cultured on stiff hydrogels. Exposure of hMSCs to a low or high mechanical dose revealed that nuclear morphology, histone acetylation, and chromatin condensation are reversible when the time of exposure to stiff substrates is short, but the effects can become irreversible with extended exposure and dosing. HAT1 appears to play a role in this (ir)reversibility as mRNA levels remain upregulated to maintain histone acetylation levels after softening. Here, we provided new insight in the role of mechanical dosing on chromatin remodeling over time which raises the possibility for the use of HAT/HDAC modulators to optimize cell expansion in vitro.

## 5 Experimental Section

*Synthesis of Hydrogel Precursors*: A complete description of all synthetic methods and molecular characterization is provided in the Supporting Information.

*Hydrogel Fabrication*: Phototunable hydrogels were prepared by copolymerizing eight‐arm PEG thiol (*M*_n_ = 20k), Maleimide allyl sulfide cross‐linker **2** and maleimide functionalized RGD **3** in dimethylformamide (DMF). A 10.1 wt% solution with a final concentration of 35.5 × 10^−3^ m PEG thiol, 39 × 10^−3^ m cross‐linker **2**, and 2.5 × 10^−3^ m maleimide RGD **3** was prepared, and polymerization was initiated by adding TEOA at a final concentration of 0.75 × 10^−6^ m. Gelation occurred after 10 min on 12 or 25 mm thiolated cover glasses with a final thickness of 100 × 10^−6^ m.

*Mechanical Properties of Hydrogels*: Oscillatory rheology was performed on a TA Instruments DHR‐3 rheometer with an 8 mm parallel plate geometry and a quartz lower plate to allow UV irradiation. Allyl sulfide crosslinked PEG hydrogels were prepared by mixing stock solutions of eight‐arm PEG thiol (*M*_n_ = 20k) and allyl sulfide dimaleimide cross‐linker in DMF to a final concentration of 40 × 10^−3^ m thiol and 44 × 10^−3^ m maleimide. The precursor solution was mixed and placed on the rheometer with the gap immediately lowered to 100 µm. Hydrogel network evolution was monitored in situ using an oscillatory shear strain of 1% and a frequency of 1 rad s^−1^ (within the linear viscoelastic range). Young's modulus was calculated with *E* = 2 × (1 + ν) × *G*′, where a Poisson's ratio (ν) of 0.5 for the PEG hydrogels was assumed. For in situ photosoftening the gel was immersed in a bath of 1.7 × 10^−3^ m of photoinitiator LAP and 12 × 10^−3^ m oxidized glutathione (Sigma, Cat. No. G4376). After 30 min, the gel was placed on the rheometer and exposed to 365 nm light (*I*_0_ = 10 mW cm^−2^) for 1 min and the change in shear storage modulus *G*′ was monitored using the same dynamic time sweep parameters. To measure the swollen modulus after softening, hydrogels softened with 6, 8, 10, or 12 × 10^−3^ m glutathione and 1.7 × 10^−3^ m LAP were compressed to 15% strain at 0.5 mm min^−1^, using a MTS Synergie 100 (10 N) at 0, 5, and 40 d after softening. The compressive modulus was estimated as the slope of the linear region of the stress–strain curve between 0 and 5% strain and reported as the Young's modulus.

*(In Situ) Modulation and Measurement of Hydrogel Properties*: To induce gel softening, stiff hydrogels were equilibrated in a solution of 1.7 × 10^−3^ m of photoinitiator LAP and 10 × 10^−3^ m oxidized glutathione in phosphate buffered saline (PBS) for 30 min. After 365 nm light exposure (*I*_0_ = 10 mW cm^−2^; Omnicure 1000, Lumen Dynamics) for 2 min. Gels were washed with PBS prior to cell seeding. For in situ photodegradation, culture media was exchanged with culture media supplemented with 1.7 × 10^−3^ m of photoinitiator LAP and 10 × 10^−3^ m oxidized glutathione and allowed to swell for 30 min. Gels were exposed to 365 nm light (*I*_0_ = 10 mW cm^−2^; Omnicure 1000, Lumen Dynamics) for 2 min to induce gel softening. Gels were washed with culture media to remove excess LAP and glutathione.

*Mesenchymal Stem Cell Isolation and Culture*: Cell culture reagents were purchased from Invitrogen, except as noted. hMSCs (18, female) were isolated from fresh human bone marrow (Lonza) following the protocol described in ref. 45. Isolated hMSCs were labeled as P1 and frozen down in Cell Freezing Medium‐DMSO 1× (Sigma, Cat. No. C6164) and stored in liquid nitrogen. P1 hMSCs were expanded in culture media (low‐glucose Dulbecco's modified Eagle media (DMEM) supplemented with 50 µg mL^−1^ penicillin, 50 µg mL^−1^ streptomycin, and 1 µg mL^−1^ fungizone) containing 20% (vol/vol) fetal bovine serum (FBS) to generate P2 cells, which were used in all the reported experiments in this manuscript. Cell seeding density on hydrogels was 3000 cells cm^−2^ for immunostaining, and 6000 cells cm^−2^ for RT‐qPCR. One day post‐seeding, hydrogel samples were transferred to a new well plate with fresh media to eliminate any confounding influence of hMSCs that attached to the TCPS instead of the hydrogels. For TSA treatment, TSA (Sigma, Cat. No. T8552) in DMSO was added to the media at a concentration of 300 × 10^−9^ m and cultured for 3 d. For blebbistatin treatment, blebbistatin (Sigma, Cat. No. B0560) in DMSO was added to the media at a concentration of 50 × 10^−6^ m 1 d after cell seeding and cultured for an additional 2 d. Samples were fixed 1, 2, 3, 4, 6, or 20 d after cell seeding.

*Immunostaining*: hMSCs were fixed in 2% paraformaldehyde for 30 min at room temperature, rinsed in PBS twice, and then permeabilized using 0.1% TritonX‐100 in PBS for 1 h. Next, samples were blocked in 5% bovine serum albumin (BSA) for 1 h at room temperature to minimize nonspecific protein binding. Anti‐YAP (1:250, mouse, Santa Cruz, CA, Cat. No. 101199), anti‐AcK (1:300, rabbit, Abcam, Cat. No. 190479), and anti‐HDAC3 (1:250, rabbit, Abcam, Cat. No. 32369) primary antibodies in 5% BSA were added to the samples and incubated for 1 h at room temperature. Primary antibodies were removed by rinsing in PBST (0.5 wt% Tween‐20 in PBS) two times for 20 min. Samples were then incubated at room temperature with secondary antibodies (1:1000, goat anti‐rabbit AlexaFluor 647, ThermoFisher, Cat. No. A21245; goat anti‐mouse Alexa‐Fluor 488, ThermoFisher, Cat. No. A11001), rhodamine phalloidin (1:300, ThermoFisher, Cat. No. R415), and DAPI (1 mg mL^−1^; Sigma, Cat. No. 10236276001) in 1% BSA. After 1 h, the secondary antibody solution was removed, and the samples were rinsed two times for 20 min with PBST. All immunostained samples were stored in PBS at 4 °C until imaging (Operetta; Perkin Elmer). Nuclei were imaged with a confocal microscope (LSM 710, Zeiss). At least 100 cells and 20 nuclei were acquired for each of three gels at all time points.

*Analysis of Cell Morphology, Nucleus Morphology, YAP Ratio, and Histone Acetylation*: Cell morphology, YAP ratio, and histone acetylation were analyzed using Harmony High Content Imaging and Analysis software (Perkin Elmer). For cell morphology, cytoplasmic outlines were identified based on F‐actin staining, respectively, using the Find Cytoplasm building block. The area and roundness were quantified by using the Calculate Morphology Properties building block. YAP ratio was quantified by first measuring the mean intensity of the YAP staining in the nucleus area and cytoplasm area using the Calculate Intensity Properties building block. In the Define Results building block, the ratio between the intensity in the nucleus area and cytoplasm area was calculated. Histone acetylation was quantified by measuring the mean intensity of histone acetylation in the nucleus area and normalized to the control condition. HDAC3 levels were quantified by measuring the mean intensity of HDAC3 in the nucleus area and normalized to the control condition. During the analyses, cell clusters were avoided and only single isolated cells and cells that barely touch boundary were analyzed. For nuclear volume and sphericity, 3D stacks were analyzed using Imaris image analysis software (Bitplane). Specifically, images were subjected to a universal threshold to obtain 3D renderings representative of the observed nuclear morphology. The sphericity of each nucleus was then calculated as the ratio of the surface area of a perfect sphere with the same volume to the measured surface area of the nucleus.

*Analysis of Chromatin Condensation*: To analyze chromatin condensation, a CCP was calculated for each nucleus based on the DAPI staining. CCP was generated using a MATLAB script from Maucks group in which a gradient‐based Sobel edge detection algorithm was used to measure the edge density for individual nuclei.^37^ The MATLAB script was adapted to analyze 18‐bit images taken with a confocal microscope (LSM 710, Zeiss).

*RNA Isolation and Quantitative Real‐Time Polymerase Chain Reaction*: qRT‐PCR was used to quantify the mRNA expression levels of HDAC1, HDAC2, HDAC3, and HAT1 relative to the reference gene for GAPDH. RNA was isolated from hMSCs on ≈1520 mm^2^ substrate 3, 6 (reversible condition), or 20 d (irreversible condition) after seeding onto the hydrogels using the RNeasy Micro Kit (Qiagen, Cat. No. 74004). RNA quantity and purity were measured via spectrophotometry (ND‐1000; NanoDrop). cDNA was synthesized from total RNA using the iScript Synthesis kit (Bio‐Rad, Cat. No. 1708841) and relative mRNA expression levels were measured via qRT‐PCR using SYBR Green reagents (Bio‐Rad, Cat. No. 1708884) on an iCycler (Bio‐Rad) and normalized to GAPDH for three technical replicates per condition. Primer sequences are listed in **Table** **1**.

**Table 1:** Primer sequences for qRT‐PCR

Gene	Forward primer (5′–3′)	Reverse primer (5′–3′)
GAPDH	GCAAGAGCACAAGAGGAAGAG	AAGGGGTCTACATGGCAACT
HDAC1	AACCTGCCTATGCTGATGCTGG	TCGTCTTCGTCCTCATCG
HDAC2	CAACGCAGCCCATTCACC	GCAAGTTATGGGTCATGCGG
HDAC3	AGTTCTGCTCGCGTTACACA	CAGAAGCCAGAGGCCTCAAA
HAT1	GCTCCCACTTGGATCTCGAC	GCACCAAATCCCTAAAAGAGAAGG

*Western Blot*: Cells were washed three times with PBS and lysed in radioimmunoprecipitation assay (RIPA) (ThermoFisher, Cat. No. 89900) buffer supplemented with 1% protease and phosphatase inhibitor cocktail (ThermoFisher, Cat. No. 78440). Protein concentrations were determined with a micro BCA kit (ThermoFisher, Cat. No. 23235). Lysates were subsequently combined with 5× Laemmli sample buffer and heated to 95 °C for 5 min. Cell lysates were separated on precast 4–12% gradient (Bio‐Rad, Cat. No. 4561094) in running buffer. Proteins were transferred to PVDF membrane (Bio‐Rad, Cat. No. 1620218) in transfer buffer (13.3 g glycine, 3.03 grams Tris‐Base, 10% methanol) for 90 min at 0.4 A and 130 V at 4 °C. Membranes were probed for total protein with REVERT total protein stain kit (LI‐COR Biosciences, Cat. No. 926‐11010) and imaged. Subsequently, membranes were blocked in TBST and 5% skim milk powder at RT for 1 h and incubated overnight in anti‐AcK (1:500, rabbit, Abcam, Cat. No. 190479) antibody in blocking solution at 4 °C. Membranes were incubated with secondary goat‐anti‐rabbit HRP conjugated antibody (1:3000, ThermoFisher, Cat. No. 65‐6120) for 1 h at RT. Chemiluminescence signal was detected using Pierce ECL Plus solution (ThermoFisher Scientific) and an ImageQuant LAS 4000 detector.

*Statistical Analysis*: All experiments were performed with at least three replicates per condition. For morphology, YAP, and histone acetylation analyses, at least 100 cells were analyzed per replicate. For nuclear morphology and CCP, at least 20 cells were analyzed per replicate. Data were compared using one‐way ANOVA and Tukey post tests or Student's *t*‐test in Prism 7 (GraphPad Software, Inc.) unless otherwise stated. Data are presented as mean ± standard deviation or as Tukey box plots with whiskers with maximum 1.5 interquartile range (IQR).

## Conflict of Interest

The authors declare no conflict of interest.

## Supporting information

Supplementary
