# Supplementary material for: Extended Exposure to Stiff Microenvironments Leads to Persistent Chromatin Remodeling in Human Mesenchymal Stem Cells
Source: Adv Sci (Weinh). 2018 Dec 10;6(3):1801483. doi: 10.1002/advs.201801483 (PMC6364489; doi:10.1002/advs.201801483)
Supplement: Supplementary file 1 — Supplementary [file ADVS-6-1801483-s001.pdf]

Copyright WILEY-VCH Verlag GmbH & Co. KGaA, 69469 Weinheim, Germany, 2018.

Supporting Information

for *Adv. Sci.,* DOI: 10.1002/advs.201801483

Extended Exposure to Stiff Microenvironments Leads to Persistent Chromatin Remodeling in Human Mesenchymal Stem Cells

*Anouk R. Killaars, Joseph C. Grim, Cierra J. Walker, Ella A. Hushka, Tobin E. Brown, and Kristi S. Anseth**

Supplemental Information

Extended Exposure to Stiff Microenvironments Leads to Persistent Chromatin Remodeling in Human Mesenchymal Stem Cells

*Anouk R. Killaars, Joseph C. Grim, Cierra J. Walker, Ella A. Hushka, Tobin E. Brown, Kristi S. Anseth**

A. R. Killaars Author 1,
Program of Materials Science and Engineering and BioFrontiers Institute, University of Colorado Boulder, Jennie Smoly Caruthers Biotechnology Building, 3415 Colorado Ave, Boulder, CO 80303, USA
Dr. J. C. Grim Author 2,
Department of Chemical and Biological Engineering and BioFrontiers Institute, University of Colorado Boulder, Jennie Smoly Caruthers Biotechnology Building, 3415 Colorado Ave, Boulder, CO 80303, USA
C. J. Walker Author 3
Program of Materials Science and Engineering and BioFrontiers Institute, University of Colorado Boulder, Jennie Smoly Caruthers Biotechnology Building, 3415 Colorado Ave, Boulder, CO 80303, USA
E. A. Hushka Author 4, T. E. Brown Author 5
Department of Chemical and Biological Engineering and BioFrontiers Institute, University of Colorado Boulder, Jennie Smoly Caruthers Biotechnology Building, 3415 Colorado Ave, Boulder, CO 80303, USA
Prof. K. S. A.
Department of Chemical and Biological Engineering and BioFrontiers Institute, University of Colorado Boulder, Jennie Smoly Caruthers Biotechnology Building, 3415 Colorado Ave, Boulder, CO 80303, USA. e-mail address: kristi.anseth@colorado.edu

Keywords: allyl-sulfide hydrogels, mesenchymal stem cells, chromatin remodeling, mechanical memory

Supporting figures cited in the main text

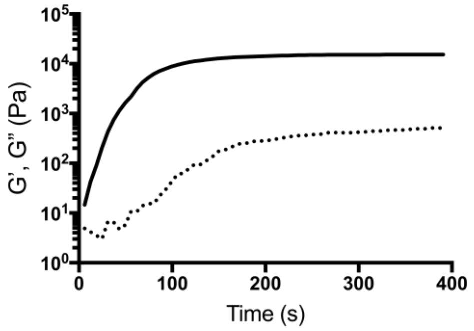

Figure S1: Allyl-sulfide gel formation. Gelation is monitored by *in situ* shear rheology and the storage modulus (solid line) and loss modulus (dotted line) were tracked.

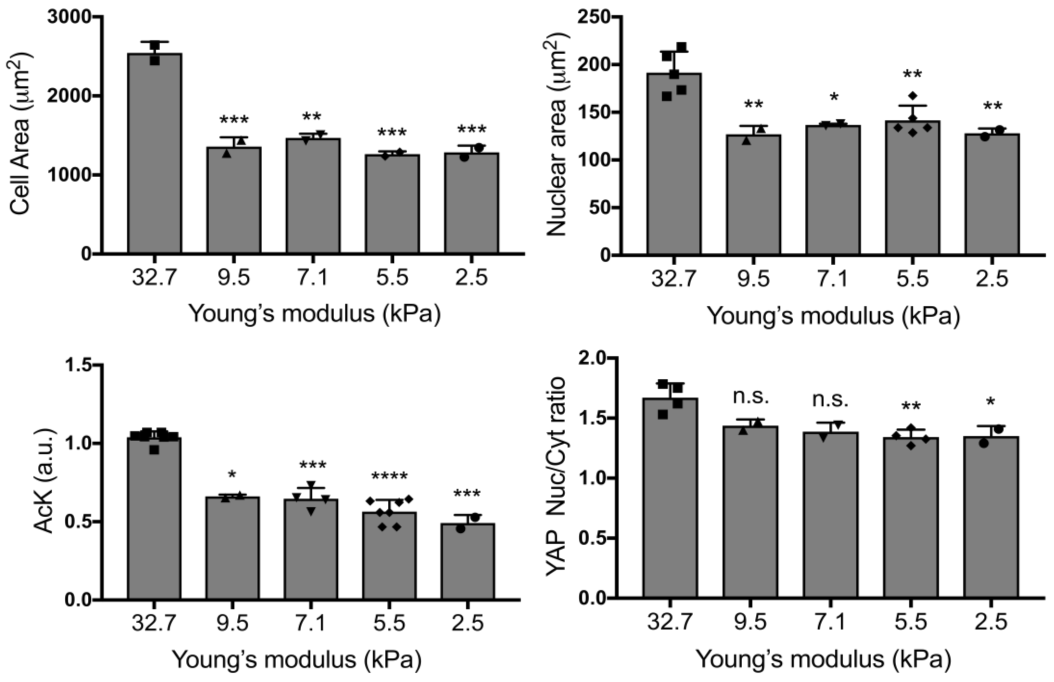

Figure S2: Influence of substrate moduli on cell morphology, nuclear morphology, YAP localization and AcK modification. Cell area, nuclear area, YAP nuclear to cytoplasmic ratio (Nuc/Cyt ratio) and AcK intensity was quantified based on immunostaining after 3 day culture. hMSCs on all soft conditions showed significant smaller cell area, nuclear area and decrease in AcK intensity. However, only soft

conditions with a Young's modulus of 5.5 and 2.5 kPa showed a significant decrease in YAP Nuc/Cyt ratio. n.s.: $p > 0.05$, *: $p < 0.05$, **: $p < 0.01$, ***: $p < 0.001$, ****: $p < 0.0001$, based on one-way ANOVA followed by Tukey's post-hoc test. n = 3 with more than 100 hMSCs analyzed per sample. The data represent the mean value ± s.d.

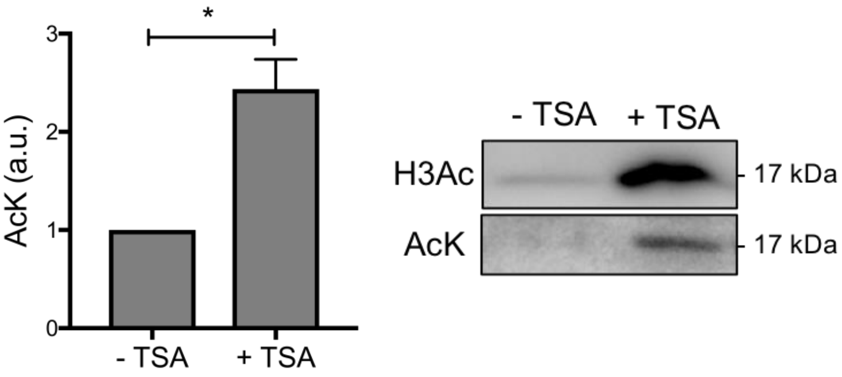

Figure S3: Verification of histone acetylation immunostaining by western blot. We cultured hMSCs on TCPS for 3 days treated with and without a common HDAC inhibitor Trichostatin A (TSA) (300 nM) and performed immunostaining and western blot subsequently. Histone acetylation was increased when treated with TSA observed for both the immunostaining and western blot. *: $p < 0.05$, based on t-test. n = 3 with more than 100 hMSCs analyzed per sample. The data represent the mean value ± s.d.

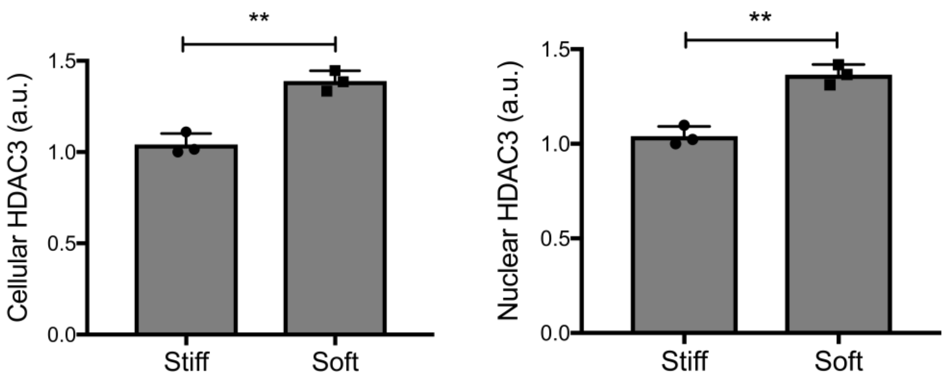

Figure S4: Influence of substrate modulus on HDAC3 levels. HDAC3 levels were quantified based on immunostaining. Global cellular HDAC3 intensity and nuclear HDAC3 intensity was significantly higher

in hMSCs cultured on soft substrates. **: $p < 0.01$, based on t-test. n = 3 with more than 100 hMSCs analyzed per sample. The data represent the mean value ± s.d.

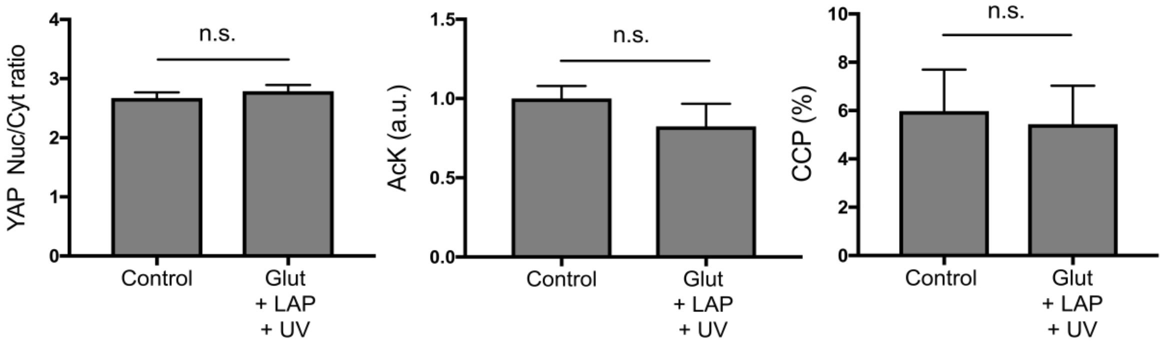

Figure S5: Influence of softening conditions on YAP localization, AcK levels and CCP. hMSCs cultured on coverslips (CS) for 1 day were incubated with glutathione (10 mM) and LAP (1.7 mM) in culture media and irradiated with UV light (I_0 = 10 mW/cm^2) for 2 min. YAP nuclear to cytoplasmic ratio (Nuc/Cyt ratio), AcK and CCP were quantified and compared to hMSCs cultured on CS with normal culture media. There was no significant difference found between the two conditions. n.s.: $p > 0.05$ based on t-test. n = 3 with more than 100 hMSCs analyzed per sample. The data represent the mean value ± s.d.

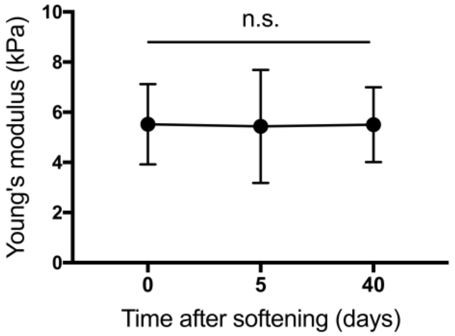

Figure S6: Characterization of Young's modulus after light-induced softening. Young's modulus right after softening, 5 days and 40 days after softening was characterized. Young's modulus does not significantly change after 40 days. n.s.: $p > 0.05$ based on one-way ANOVA followed by Tukey's post-hoc test. The data represent the mean value ± s.d.

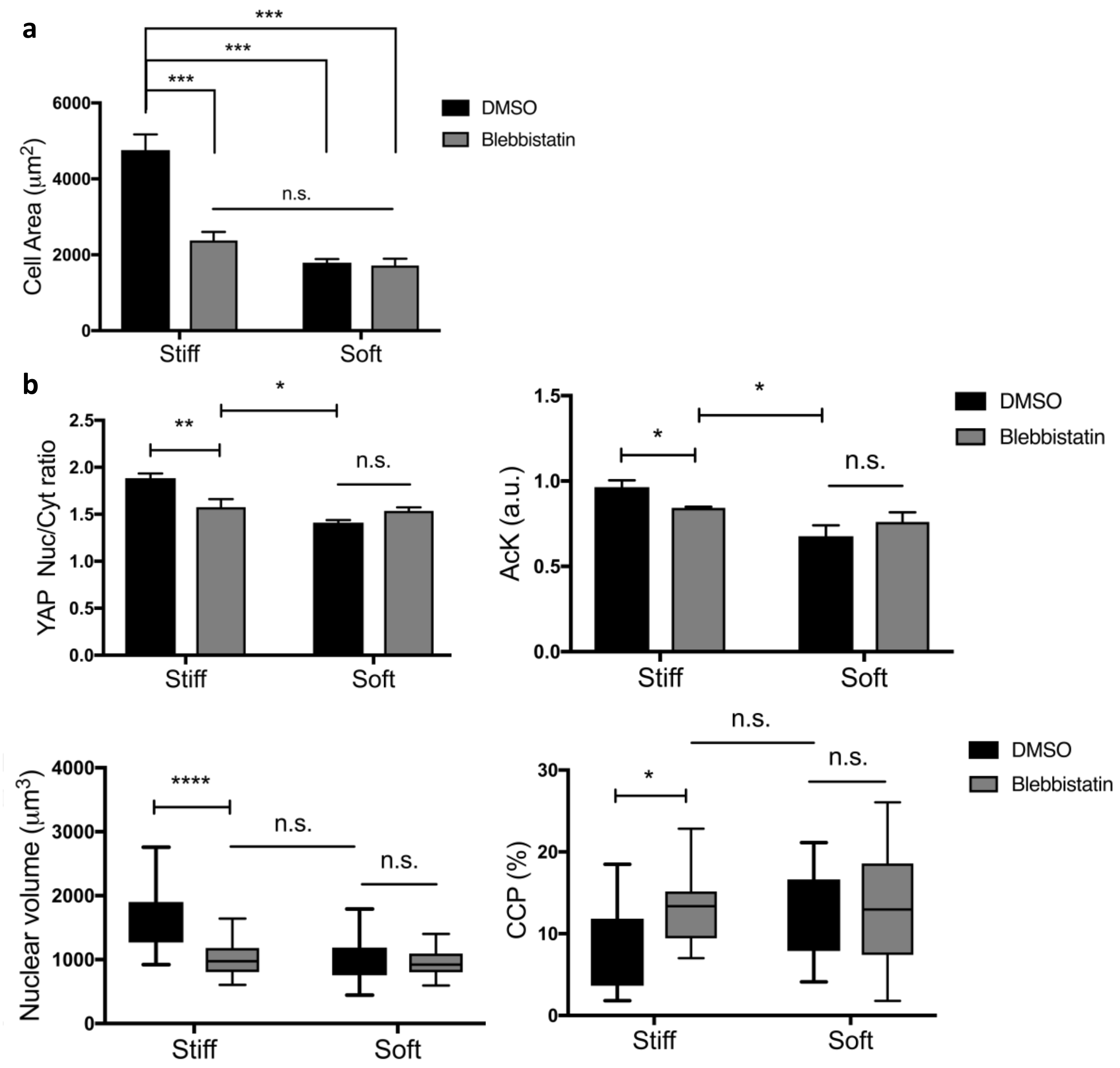

Figure S7: Influence of disruption of cytoskeletal tension on chromatin remodeling. hMSCs were cultured on stiff and soft substrates for 24 hours and subsequently treated with and without blebbistatin (50 μM in DMSO) for 48 hours. **a)** Cytoskeletal tension was efficiently disrupted as seen by cell area. **b)** YAP Nuc/Cyt ratio and histone acetylation on stiff treated with blebbistatin decreases to levels between the stiff and soft DMSO control. Nuclear volume on stiff treated with blebbistatin decreases to soft basal levels, while chromatin condensation in hMSCs cultured on stiff substrates treated with blebbistatin increases to soft basal levels. n.s.: $p > 0.05$ *: $p < 0.05$, **: $p < 0.01$, ****: $p < 0.0001$, based on a two-

way ANOVA followed by Tukey's post-hoc test. $n \geq 3$ with more than 100 hMSCs analyzed per sample. Boxplots: values are shown as median ± 1.5 IQR. Barplots: the data represent mean value ± s.d.

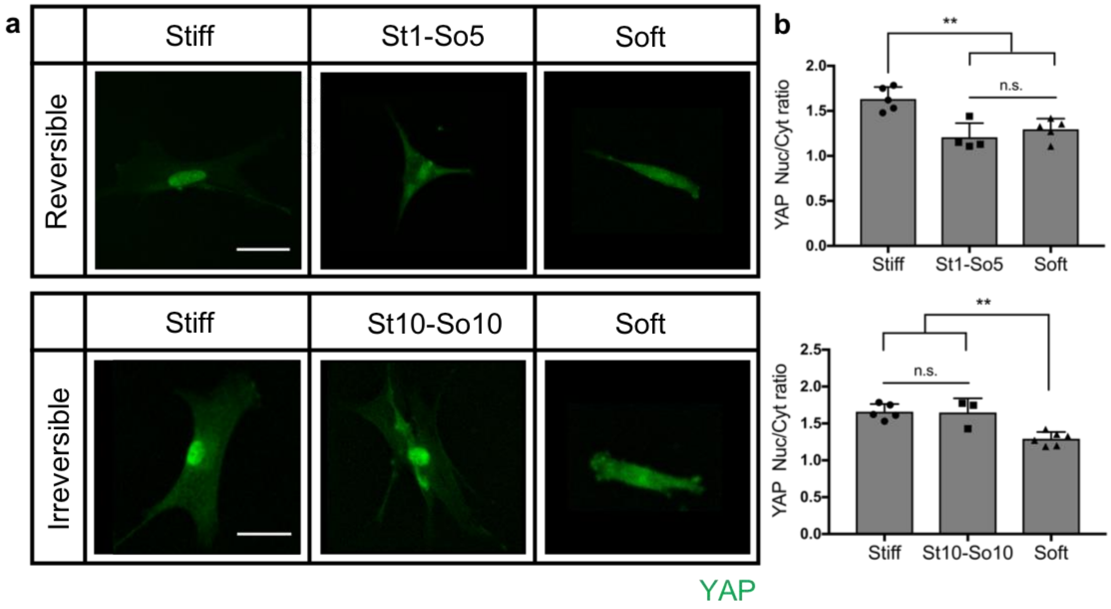

Figure S8: Influence of reversible and irreversible mechanical dosing on YAP localization. a) Immunostaining of hMSCs cultured in a reversible or irreversible condition with their stiff and soft control conditions. hMSCs cultured in the reversible condition (St1-So5) showed cytoplasmic YAP localization, similar to the soft control. hMSCs cultured in the irreversible condition (St10-So10) showed nuclear YAP localization, similar to the stiff control. YAP (green). Scale bars = 50 µm. **b)** YAP nuclear to cytoplasmic ratio (Nuc/Cyt ratio) was quantified based on immunostaining for both reversible and irreversible conditions. Softening after 1 day (St1-So5 condition) allowed YAP to diffuse back to the cytoplasm, showing a YAP Nuc/Cyt ratio similar to the soft condition. Softening after 10 days (St10-So10 condition) resulted in persistent YAP nuclear localization, showing a YAP Nuc/Cyt ratio similar to the stiff condition. n.s.: $p > 0.05$, **: $p < 0.01$ based on a one-way ANOVA followed by Tukey's post-hoc test. $n \geq 3$ with more than 100 hMSCs analyzed per sample. The data represent the mean value ± s.d.

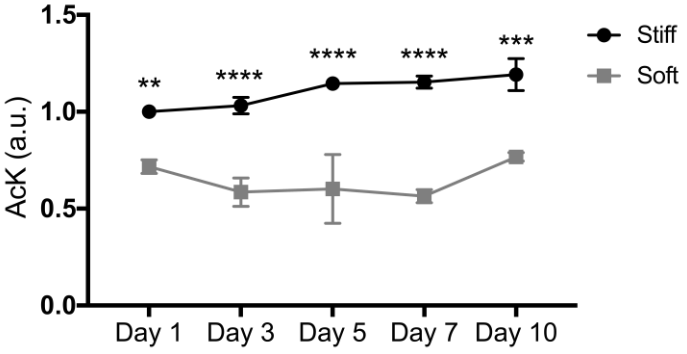

Figure S9: AcK modification in hMSCs cultured on stiff and soft substrates over time. AcK levels are significantly higher in hMSCs cultured on stiff compared to soft at all time points. ****, p < 0.0001 based on a one-way ANOVA followed by Tukey's post-hoc test. n = 250 cells per condition. The data represent the mean value ± s.d.

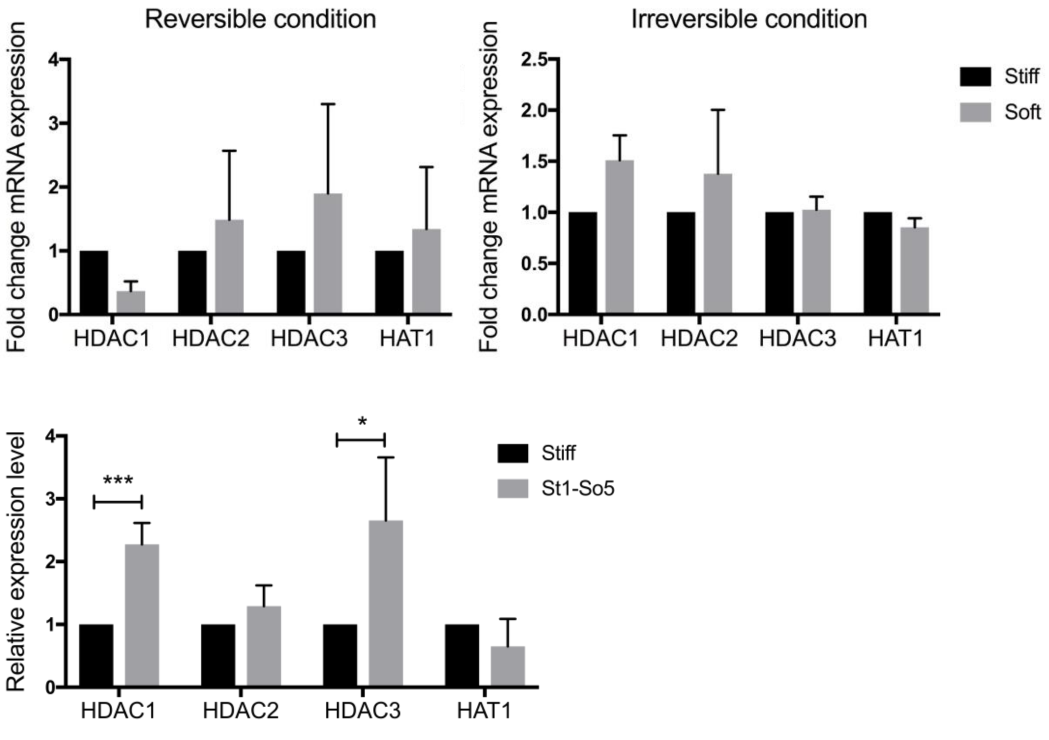

Figure S10: Influence of reversible and irreversible mechanical dosing on mRNA expression of epigenetic modulators on stiff and soft controls. RT-qPCR was performed and mRNA expression in the soft condition was normalized to mRNA expression in the stiff condition. Expression of HDAC1,

HDAC2 and HDAC3 is increased in hMSCs cultured on reversible soft (6 day culture) and irreversible soft (20 day culture), whereas HAT1 expression was decreased in hMSCs cultured on reversible soft and irreversible soft. mRNA expression of HDAC1 and HDAC3 is increased in the St1-So5 condition compared to the stiff control condition. *: $p < 0.05$, ***: $p < 0.001$, based on a one-way ANOVA followed by Tukey's post-hoc test. n = 3 with triplicates. The data represent the mean value ± s.d.

General synthetic methods

All moisture- and oxygen-sensitive reactions were carried out in flame-dried glassware under a nitrogen atmosphere. Unless otherwise noted, all reagents and solvents were the highest commercially available grades and used without further purification. All chemicals were purchased from Sigma Aldrich unless noted otherwise methanol (MeOH) and methylene chloride (CH_2Cl_2) were purified via a solvent purification system (Innovative Technologies) and water (H_2O) was purified with a MilliQ purification system (Millipore). Analytical thin layer chromatography (TLC) was used to monitor reactions and was performed on 0.25 mm pre-coated Silica Gel 60 F254 (Merck). Compounds were visualized with ultraviolet light (254 nm) and/or charring with *p*-anisaldehyde (15 g *p*-anisaldehyde, 5 mL H_2SO_4, 1 mL AcOH, 250 mL ethanol). Flash chromatography was performed on 230 – 400 mesh SiliaFlash® P60 silica gel (Silicycle).

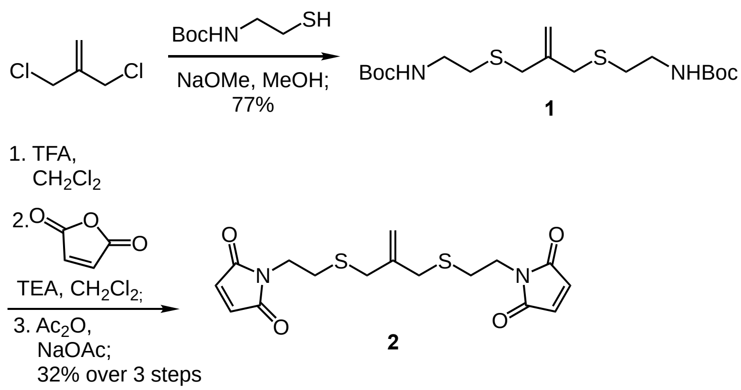

Scheme S1. Synthesis of maleimide allyl sulfide crosslinker.

^{1}H and ^{13}C nuclear magnetic resonance (NMR) spectra were recorded on Bruker AV-III 400 MHz spectrometer. Chemical shifts were reported relative to residual solvent peaks in parts per million ($CHCl_3$: ^{1}H δ

7.26). Peak multiplicity is reported as singlet (s), doublet (d), multiplet (m), doublet of doublet (dd), etc. All spectra are reported in the supplemental information. High resolution electrospray ionization mass spectra (HRESI-MS) were obtained on a Thermo Finnigan LTQ Orbitrap (electrospray ionization, time-of-flight analyzer). Peptide analysis was performed with α-cyano-4-hydroxycinnamic acid (CHCA) matrix on a Voyager DE-STR MALDI-TOF (matrix-assisted laser desorption ionization, time-of-flight analyzer).

3-((2-boc-amino)ethanethiol)-2-((2-boc-amino)ethanethiol)methyl-1-propene 1. To a 3-necked round-bottom flask was added 2-(boc-amino)ethanethiol (3.12 g, 17.6 mmol) in MeOH (10 mL). A 5.4 M NaOMe solution in MeOH (5.9 mL, 32.0 mmol)) was added. A solution of 3-chloro-2-chloromethyl-1-propene (0.926 mL, 8.0 mmol) in MeOH (10 mL) was added dropwise over 20 min via addition funnel. A white precipitate formed. The solution was heated to 60°C and stirred overnight. The reaction mixture was diluted with water (100 mL) and extracted with CH_2Cl_2 (3 x 100 mL). The combined organic layers were washed with brine, dried with $MgSO_4$, filtered, and concentrated under reduced pressure. The crude oil was purified via automated flash chromatography (10 – 80% EtOAC in hexanes) to afford **1** as a white solid (2.50 g, 6.16 mmol, 77% yield). ^{1}H NMR (400 MHz, $CDCl_3$) δ 5.04 (s, 2H), 4.12 (q, J = 7.1 Hz, 1H), 3.30 (s, 6H), 2.55 (t, J = 6.6 Hz, 4H), 2.04 (s, 1H), 1.44 (s, 19H), 1.26 (t, J = 7.1 Hz, 1H). HRMS (EMM): calculated for $C_{18}H_{34}N_2O_4S$ $[M+Na]^+$: 407.2033, found 407.2038.

Maleimide allyl sulfide crosslinker 2. To a solution of **1** (2.4 g, 5.9 mmol) in CH_2Cl_2 (10 mL) was added trifluoroacetic acid (TFA) (5 mL). The solution was stirred for 2.5 h and concentrated under reduced pressure to afford a clear oil. The oil was dissolved in CH_2Cl_2 (47 mL). Triethylamine (2.4 mL, 21.3 mmol) and maleic anhydride (2.09 g, 21.3 mmol) were added. The

suspension was stirred for 4 h. The suspension was filtered, and the filtrate was washed with CH_2Cl_2 and 1M HCl. The filtrate was dried under vacuum overnight. The filtrate was dissolved in acetic anhydride (3.9 mL, 41.81 mmol). Sodium acetate (0.34 g, 4.18 mmol) was added, and the suspension was heated to 100°C for 3 h. After cooling to room temperature, saturated $NaHCO_3$ (150 mL) was added. After bubbling ceased, the mixture was extracted with CH_2Cl_2 (2 x 100 mL). The combined organic layers were washed with brine, dried with $MgSO_4$, filtered, and concentrated under reduced pressure. The crude material was purified by automated flash chromatography (10 – 100% EtOAc in hexanes) to afford **2** was a white solid (0.69 g, 1.88 mmol, 32% over 3 steps). ^1H NMR (400 MHz, $CDCl_3$) δ 6.72 (s, 4H), 5.19 – 4.91 (m, 2H), 3.68 (dd, *J* = 7.5, 6.5 Hz, 5H), 3.29 (t, *J* = 0.6 Hz, 5H), 2.81 – 2.47 (m, 4H). HRMS (EMM): calculated for $C_{16}H_{18}N_2O_4S_2$ $[M+LI]^+$: 373,0868, found 373.0868.

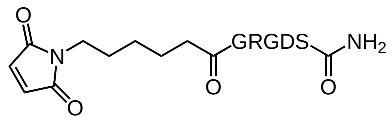

3

Maleimido-Gly-Arg-Gly-Asp-Ser-CONH$_2$ 3. Peptide synthesis was carried out on Rink Amide MBHA resin (EMD Millipore) using standard HATU/Fmoc coupling chemistry conditions on a Protein Technologies Tribute Peptide Synthesizer. After synthesis of the Resin-Ser-Glu-Gly-Asp-Gly-NH_2 peptide, 6-maleimidohexanoic acid was coupled to the *N*-terminus manually. To a vial containing 6-maleimidohexanoic acid (0.422 g, 2 mmol) was added HATU (0.76 g, 2.0 mmol) and DMF (6 mL). DIEA (0.69 mL, 4.0 mmol) was added and the yellow solution was transferred to a reaction vial with a fritted filter containing the resin (0.50 mmol). The solution was stirred for 4 h. The solvent was filtered, and the resin was washed with DMF (3x), and CH_2Cl_2 (3x). The resin tested negative for free amines via the Kaiser Ninhydrin test. To cleave the peptide from the resin, the resin was swelled for 30 min in CH_2Cl_2. A solution of 88:5:5:2 TFA:phenol:H_2O:triisopropyl silane (6 mL) was added. The suspension was stirred for 1 h. The

filtrate was collected, and the resin was washed with TFA (2x). The combined TFA washes were concentrated to ~1 mL by gently blowing air over the solution. The peptide was precipitated into ice cold diethyl ether (45 mL) and pelleted via centrifugation (15 mins at 3.0 rcf). The peptide was purified by high performance liquid chromatography (HPLC) on a Waters 2545 HPLC equipped with an XSELECT CSH Prep C18 column using a 20:80 ACN:H_2O → 95:5 ACN:H_2O gradient. MALDI-TOF analysis confirmed the mass of peptide **3**.

NMR Spectra

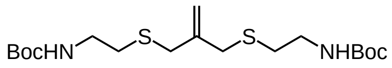

1

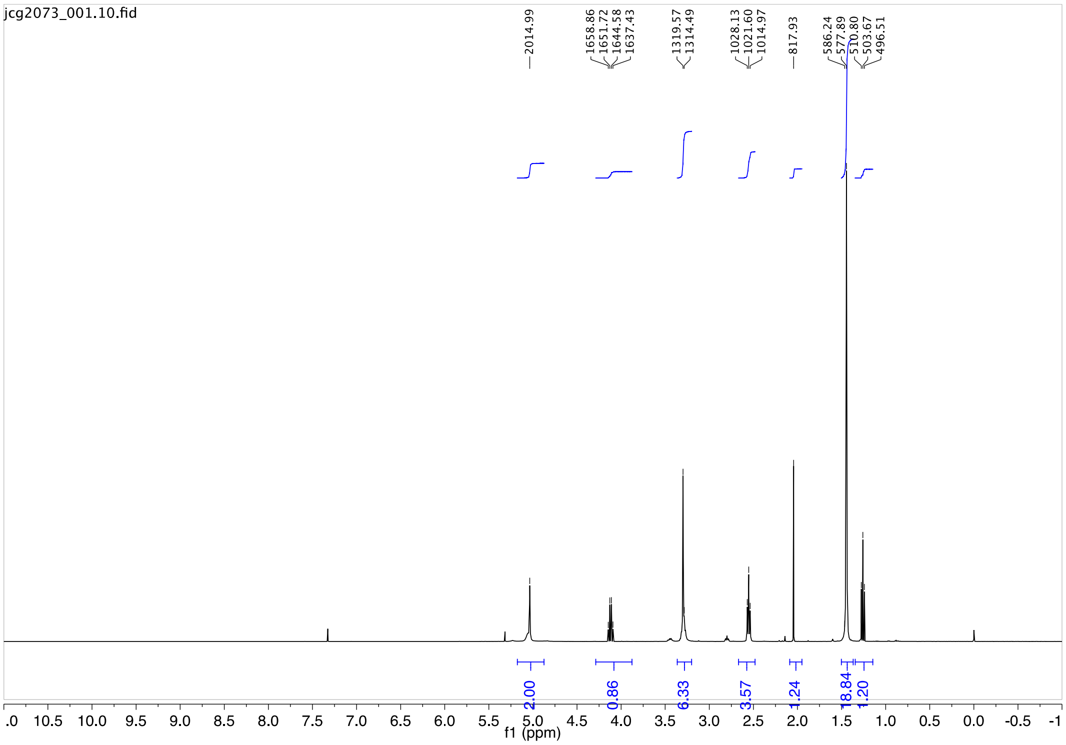

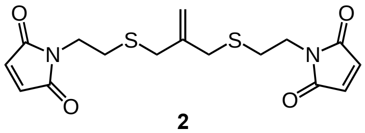

2

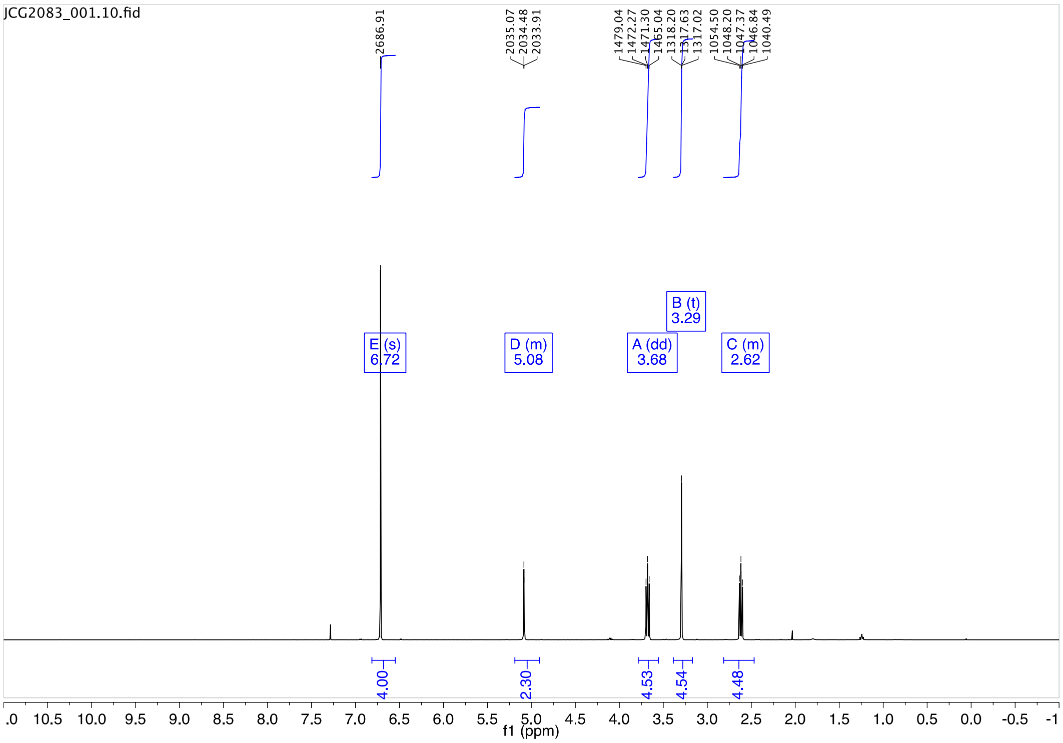

JCG2083_001.10.fid
2686.91
2035.07
2034.48
2033.91
1479.04
1472.27
1471.30
1465.04
1318.20
1317.63
1317.02
1054.50
1048.20
1047.37
1046.84
1040.49
E (s)
6.72
D (m)
5.08
A (dd)
3.68
B (t)
3.29
C (m)
2.62
4.00
2.30
4.53
4.54
4.48
f1 (ppm)
